# Control of the noncollinear interlayer exchange coupling

**DOI:** 10.1126/sciadv.abd8861

**Published:** 2020-11-25

**Authors:** Zachary R. Nunn, Claas Abert, Dieter Suess, Erol Girt

**Affiliations:** 1 Simon Fraser University, 8888 University Drive, Burnaby, British Columbia V5A 1S6, Canada.; 2 Faculty of Physics, University of Vienna, Austria.; 3 University of Vienna Research Platform MMM Mathematics - Magnetism - Materials, University of Vienna, Austria.

## Abstract

Interlayer exchange coupling in transition metal multilayers has been intensively studied for more than three decades and is incorporated into almost all spintronic devices. With the current spacer layers, only collinear magnetic alignment can be reliably achieved; however, controlling the coupling angle has the potential to markedly expand the use of interlayer exchange coupling. Here, we show that the coupling angle between the magnetic moments of two ferromagnetic layers can be precisely controlled by inserting a specially designed magnetic metallic spacer layer between them. The coupling angle is controlled solely by the composition of the spacer layer. Moreover, the biquadratic coupling strength, responsible for noncollinear alignment, is larger than that of current materials. These properties allow for the fabrication and study of not yet realized magnetic structures that have the potential to improve existing spintronic devices.

## Teaser

Metallic spacer layers enable precise control of the angle between the magnetic moments of two ferromagnetic layers.

## INTRODUCTION

Interlayer exchange coupling between two ferromagnetic layers across a spacer layer has been intensively investigated since the 1980s. It was discovered that the interlayer exchange coupling across most 3*d*, 4*d*, and 5*d* nonmagnetic metallic spacer layers oscillates between antiferromagnetic and ferromagnetic as a function of spacer layer thickness (*1*–*4*).

This discovery enabled the control of antiferromagnetic coupling between two ferromagnetic films, which is now used in most spintronic devices (*5*). It was also found that interlayer exchange coupling across magnetically polarizable metallic spacer layers, Pd and Pt (*6*), is ferromagnetic. Unfortunately, no spacer layer material has been identified that enables the control of noncollinear coupling, until now.

In this work, we show that noncollinear alignment can be precisely controlled if two ferromagnetic layers are coupled across a spacer layer consisting of a nonmagnetic material (Ru) alloyed with a ferromagnetic material (Fe).

**Fig. 1:**
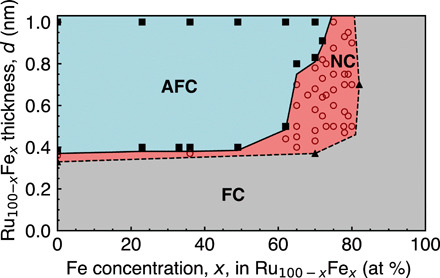
Coupling phase diagram. Phase diagram shows ferromagnetic (FC), antiferromagnetic (AFC), and noncollinear (NC) coupling regions of Co ∣ Ru_100−*x*_Fe*_x_*(*d*)∣ Co as a function of Ru_100−*x*_Fe*_x_* thickness *d* and Fe concentration in the Ru_100−*x*_Fe*_x_* spacer layer, *x*. Solid squares, empty circles, and solid triangles represent experimentally measured antiferromagnetic, noncollinear, and ferromagnetic structures, respectively. The solid and dashed lines and shaded regions are a guide to the eye.

This has not yet been observed because noncollinear coupling occurs at very high concentrations of the ferromagnetic elements in the spacer layer for which one would expect to observe ferromagnetic coupling. As is apparent from our experimental phase diagram, Fig. 1, for concentrations of Fe, *x*, below 60 atomic % (at %) in the Ru_100−*x*_Fe*_x_* spacer layer, the transition between ferromagnetic coupling and antiferromagnetic coupling is very sharp as a function of spacer layer thickness. However, the noncollinear coupling region markedly expands for *x* > 60, allowing for control of the noncollinear coupling angle. The noncollinear coupling coincides with the advent of magnetic order in the spacer layer, which is highly unexpected and cannot be explained using the current interlayer exchange coupling framework.

**Fig. 2:**
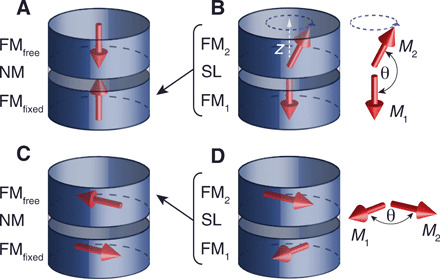
Possible noncollinear magnetic structures for device applications. Simplified schematics of nanopillars with two ferromagnetic layers (FM_fixed_ and FM_free_) separated by a nonmagnetic layer (NM) used in (**A**) the STT MRAM and (**C**) magnetic sensors. (**B** and **D**) Simplified schematics of nanopillars with two ferromagnetic layers (FM_1_ and FM_2_) coupled across our spacer layer (SL) to achieve noncollinear alignment. The noncollinear coupling structure in (B) can replace FM_fixed_ in (A) to increase the STT on FM_free_ in the STT MRAM. The noncollinear coupling structure in (D) can replace FM_free_ in (C) to fabricate magnetic sensors. The structures in (B) and (D) can be obtained by controlling the coupling across SL and the magnetic anisotropy of FM_1_ and FM_2_.

Our new spacer layers have the potential to be used in most spintronic devices, as the optimal design of these devices almost always requires noncollinear alignment between at least two adjacent ferromagnetic layers (*7*–*10*). In spin-transfer torque magnetic random access memory (STT MRAM) devices (*11*), information is stored in nanopillars consisting of two ferromagnetic layers (FM_fixed_ and FM_free_) separated by a thin nonmagnetic layer (NM), with the simplified structure shown in Fig. 2A. In these nanopillars, an electrical current is used to manipulate the magnetic moment orientation of FM_free_ due to the STT effect (*12*). The magnitude of the STT is proportional to the double cross product ${\hat{M}}_{\text{free}}\times ({\hat{M}}_{\text{fixed}}\times {\hat{M}}_{\text{free}})$, where ${\hat{M}}_{\text{fixed}}$ and ${\hat{M}}_{\text{free}}$ are the unit vectors of the magnetic moments of FM_fixed_ and FM_free_, respectively (*12*). Because of the inability to control noncollinear coupling, the optimal three-layer magnetic structure of the nanopillar is when both ferromagnetic layers have perpendicular anisotropy (Fig. 2A) (*13*). In this case, ${\hat{M}}_{\text{fixed}}$ and ${\hat{M}}_{\text{free}}$ are parallel and the STT is zero. This limits the performance of these devices since thermal fluctuations or external magnetic fields are relied upon to provide noncollinear alignment and create nonzero STT.

Our new spacer layers (SL) can be used to control the angle θ between the magnetic moments of ferromagnetic layers FM_1_ and FM_2_ in FM_1_∣SL∣FM_2_ (Fig. 2B) and hence create the desired noncollinear alignment. The structure in Fig. 2B can replace FM_fixed_ in FM_fixed_∣NM∣FM_free_ (Fig. 2A) to combine the control of the magnetization direction of FM_1_∣SL∣FM_2_ with large magnetoresistance of FM_fixed_∣NM∣FM_free_ structures. Macrospin calculations show that STT MRAM devices with noncollinear designs will have substantially improved energy efficiency (*8*, *9*).

Another thin-film magnetic device frequently used in applications is the magnetic sensor with the simplified film structure in Fig. 2C. The highest sensitivity in these magnetic sensors is obtained when the angle between the magnetic moments of ferromagnetic layers FM_fixed_ and FM_free_ is 90^∘^. In sensors with both of these ferromagnetic layers having in-plane magnetization, the noncollinear orientation is achieved by biasing FM_fixed_ along one direction with an antiferromagnetic layer and applying an external magnetic field to rotate FM_free_. With our new spacer layers, one can achieve, without applying an external field, the desired noncollinear orientation by replacing FM_free_ in Fig. 2C with the noncollinear structure FM_1_∣SL∣FM_2_ in Fig. 2D. Our proposed sensor structure also requires two antiferromagnetic layers, one biasing FM_fixed_ and the other biasing FM_2_ along the same in-plane direction. In spin-torque nano-oscillator (STNO) (*14*) and spin-orbit torque MRAM (SOT-MRAM) devices (*15*), using noncollinear magnetization alignment can also eliminate the need for an external magnetic field. These examples demonstrate that the ability to control noncollinear coupling can be of great benefit to magnetic devices. Furthermore, studying these new noncollinear structures can deepen our understanding of magnetic interface phenomena such as STT and spin pumping.

**Fig. 3:**
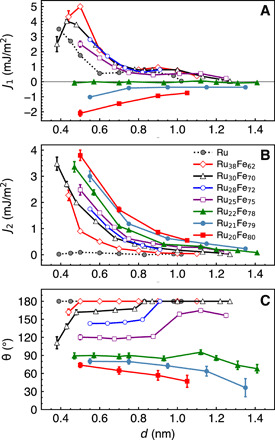
Coupling parameters as a function of spacer layer thickness. (**A**) *J*_1_, (**B**) *J*_2_, and (**C**) θ of Co(2)∣Ru_100−*x*_Fe*_x_*(*d*)∣Co(2) for 0.4 ≤ *d* ≤ 1.4 nm. Data were collected at room temperature (298 K). The noncollinear coupling region can also be visualized by plotting *J*_2_ versus |*J*_1_|, as shown in Supplemental Data 1.

In this work, controllability of the noncollinear coupling strength and angle was studied in a Co (2)∣Ru_100−*x*_Fe*_x_*(*d*)∣Co(2) trilayer by varying *x* and *d* (Fig. 3). In these structures, the numbers in parentheses indicate the layer thicknesses in nanometers, *x* is the atomic concentration of Fe in the RuFe layer, and *d* is the thickness of the RuFe layer. We chose Co layers to be only 2 nm thick to be able to precisely measure the contribution of RuFe to the total magnetic moment of Co∣RuFe∣Co. RuFe spacer layers were selected because Fe forms a solid solution with Ru over a large composition range (*16*). In addition, Co∣Ru∣Co is important for applications since it has one of the largest reported antiferromagnetic couplings (*2*).

## RESULTS

### Noncollinear coupling parameters

Shown in Fig. 3 are the bilinear (*J*_1_) and biquadratic (*J*_2_) coupling constants and zero field coupling angle (θ) between the magnetic moments of the ferromagnetic layers of Co(2)∣Ru_100−*x*_Fe*_x_*(*d*)∣Co(2). The thickness of the RuFe is 0.4 ≤ *d* ≤ 1.4 nm, with an Fe concentration of *x* = 0 and 62 ≤ *x* ≤ 80. The values of *J*_1_, *J*_2_, and θ are determined by fitting the *M*(*H*) measurements of Co∣RuFe∣Co with the one-dimensional micromagnetic model proposed by Eyrich *et al*. (*17*) and assuming that the saturation magnetization *M*_s_ and exchange stiffness, *A*_ex_, do not vary across the Co layers. In this model, the interlayer exchange coupling energy is described as

$$E_{\text{coupling}}=J_{1}\text{cos}\;(\theta )+J_{2}{\text{cos}}^{2}(\theta )$$ (1)

where *J*_1_ accounts for the strength of antiferromagnetic coupling (θ = 180^∘^) if *J*_1_> 0 and ferromagnetic coupling (θ = 0^∘^) if *J*_1_< 0. *J*_2_ is always positive in our structures and accounts for the strength of orthogonal coupling (θ = 90^∘^). The emphasis of this work is on noncollinear coupling that occurs when *J*_2_ >∣*J*_1_∣/2. More information on the fitting procedure, including examples of fitted *M*(*H*) data and Kerr microscopy measurements showing that the magnetization reversal in our films is uniform over the magnetic field range from 0.005 to 5 T, can be found in Modeling of *M*(*H*) and Kerr microscopy section in Materials and Methods.

As shown in Fig. 3, the interlayer coupling of Co layers across Ru in Co∣Ru(*d*)∣Co is antiferromagnetic (θ = 180^∘^) for 0.4 ≤ *d* ≤ 1.02 nm. *J*_1_ of Co∣Ru(*d*)∣Co oscillates and decreases with increasing *d*, in agreement with a previous report (*18*), while *J*_2_ is small in the studied *d* range. The transition from antiferromagnetic to ferromagnetic coupling in Co∣Ru_100−*x*_Fe*_x_*∣Co occurs for *x* between 62 and 82. In this RuFe composition range, *J*_1_ strongly decreases with increasing Fe concentration and becomes negative for *x* > 78. Concurrently, *J*_2_ increases with *x*, satisfying the condition required for noncollinear alignment (*J*_2_ >∣*J*_1_∣/2) between the magnetic moments of Co layers. The largest measured *J*_2_ in Co∣RuFe∣Co is larger than any *J*_2_ value previously reported (*19*–*22*).

We would like to turn the reader’s attention to Fig. 3C, in the thickness region 0.5 ≤ *d* ≤ 0.8 nm. Within this region, θ remains constant with *d* for most of the RuFe alloys. However, varying the concentration of Fe in Ru allows for precise control of θ between 180^∘^ and 90^∘^. The rate of change of θ with *x* is 7.5^∘^ per 1 at % of Fe. From a fabrication point of view, it is very important that a single parameter controls θ and that θ varies slowly with *x*.

### Magnetic properties of the spacer layer

**Fig. 4:**
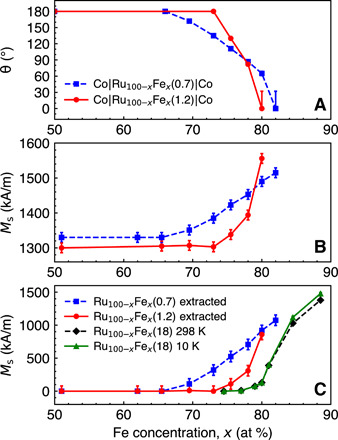
Saturation magnetization of RuFe spacer layers in noncollinear structures. (**A**) θ and (**B**) *M*_s_ of Co(2)∣Ru_100−*x*_Fe*_x_*(0.7)∣Co(2) and Co(2)∣Ru_100−*x*_Fe*_x_*(1.2)∣Co(2) at 298 K. (**C**) Extracted *M*_s_ of Ru_100−*x*_Fe*_x_*(0.7) and Ru_100−*x*_Fe*_x_*(1.2) at 298 K and measured *M*_s_ of Ru_100−*x*_Fe*_x_*(18) at 10 and 298 K.

To understand how noncollinear coupling relates to the magnetic moment of the spacer layer, three different structures were studied: Ru_100−*x*_Fe*_x_*(18) single films and Co∣Ru_100−*x*_Fe*_x_*(0.7)∣Co and Co∣Ru_100−*x*_Fe*_x_*(1.2)∣Co multilayers. Figure 4 (A and B) shows θ and *M*_s_ of Co∣Ru_100−*x*_Fe*_x_*(0.7)∣Co and Co∣Ru_100−*x*_Fe*_x_*(1.2)∣Co. Figure 4C compares the magnetic properties of the Ru_100−*x*_Fe*_x_*(18) layers to the magnetic properties of Ru_100−*x*_Fe*_x_*(0.7) and Ru_100−*x*_Fe*_x_*(1.2), which were extracted from the total *M*_s_ of Co∣Ru_100−*x*_Fe*_x_*(0.7)∣Co and Co∣Ru_100−*x*_Fe*_x_*(1.2)∣Co in Fig. 4B. Extended *M*_s_ data of Co(2)∣Ru_100−*x*_Fe*_x_*(0.7)∣Co(2) and Co(2)∣Ru_100−*x*_Fe*_x_*(1.2)∣Co(2) for 0 ≤ *x* ≤ 88 at 298 K are presented in Supplemental Data 2.

The *M*_s_ of Co∣Ru_100−*x*_Fe*_x_*(0.7)∣Co and Co∣Ru_100−*x*_Fe*_x_*(1.2)∣Co was found to increase for 0 ≤ *x* ≤ 30 and stay constant for 36 ≤ *x* ≤ 65 (Supplemental Data 2 and Fig. 4B). This initial increase in *M*_s_ is not associated with the magnetization of the RuFe layer but is due to the increase in *M*_s_ of the interface atoms of the Co layers. It has been shown that, in Co∣Ru multilayers, Co atoms at the Ru interface have a reduced magnetic moment (*17*). Adding Fe to Ru is thus expected to modify the electronic environment of the ferromagnetic Co layer’s interface atoms and increase their magnetization. For *x* > 60 in Fig. 4B, a sharp increase in *M*_s_ occurs, coinciding with the onset of noncollinear coupling (Fig. 4A). This increase in *M*_s_ is attributed to a magnetic order in the RuFe spacer layer. For 18-nm-thick Ru_100−*x*_Fe*_x_*, there is an increase in *M*_s_ for *x* ≥ 79, concurrent with the nonmagnetic-to-magnetic transition. The magnetic transition of 0.7- and 1.2-nm-thick Ru_100−*x*_Fe*_x_* in Co∣RuFe∣Co occurs at lower *x* because of the proximity polarization effect at the Co∣RuFe interfaces. In addition, the nonmagnetic-to-magnetic transition is broader and starts at lower *x* if the Ru_100−*x*_Fe*_x_* spacer layer is thinner, as would be expected in the case of the proximity polarization.

It is important to point out that these RuFe spacer layers can have large *M*_s_ values. The orthogonal alignment (θ = 90^∘^) in a Co∣Ru_100−*x*_Fe*_x_*(0.7)∣Co structure was achieved across a spacer layer with *M*_s_ = 700 kA/m. This is a larger *M*_s_ than that of ferromagnetic Ni (488 kA/m) (*23*). This is the first demonstration of noncollinear coupling occurring across a magnetic layer.

## DISCUSSION

We will now discuss the origins of the large *J*_2_ in our film structures, as *J*_2_ is responsible for the noncollinear alignment of the magnetic layers. *J*_2_ can arise from intrinsic and extrinsic sources (*4*). In the studied structures, the measured *J*_2_ always favors a perpendicular alignment and has a strength comparable to *J*_1_, suggesting an extrinsic source (*4*). Extrinsic sources of *J*_2_ could be uncorrelated film roughness (*4*), pinholes (*24*), loose spins (*25*), and spatial fluctuations (*26*). The first three are found to have negligible effect on *J*_2_, as discussed in Supplemental Data 3 and 4.

**Fig. 5:**
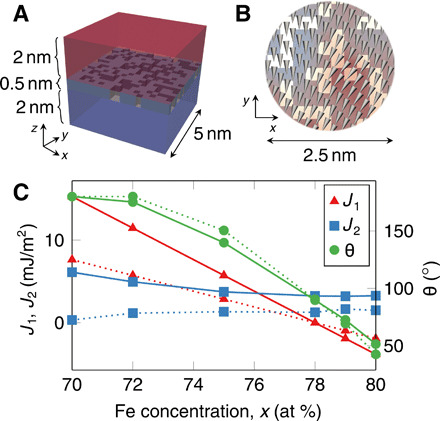
Micromagnetic simulations of noncollinear coupling. (**A**) Finite-element model of 5 nm by 5 nm Co∣RuFe∣Co with RuFe divided into ferromagnetic and antiferromagnetic regions. (**B**) Cutout of magnetization configuration in one of the magnetic layers for *x* = 78. The ferromagnetically coupled regions are marked by the dark coloring. The cutout is colored with blue and red to illustrate the inhomogeneity of the magnetization across the surface. (**C**) Simulated *J*_1_, *J*_2_, and θ versus *x* for *A*_ex_RuFe = 21 pJ/m (solid line) and *A*_ex_RuFe = 10.5 pJ/m (dotted line).

The spatial fluctuation mechanism (*26*) is based on a magnitude change of *J*_1_ across the film’s plane. To better understand how spatial fluctuations induce noncollinear coupling, we perform micromagnetic simulations with our finite-element software magnum.fe (*27*) on a 5 nm by 5 nm magnetic trilayer structure with Co, RuFe, and Co layer thicknesses of 2, 0.5, and 2 nm, respectively (Fig. 5A). The top and bottom Co layers are modeled as single-phase magnetic regions with *M*_s_ = 1.21 × 10^6^A/m and *A*_ex_(Co) = 15 pJ/m (*17*). The demagnetization field is modeled as an easy-plane anisotropy. Considering the perpendicular magnetocrystalline anisotropy of the Co layers to be *K_u_* = 2.1 × 10^5^ J/m (*17*), one can calculate the overall anisotropy as $K=K_{u}-\mu_{0}M_{s}^{2}/2=-7.1\times 10^{5}\;J/m$.

The middle RuFe spacer layer is divided into ferromagnetically and antiferromagnetically coupled regions by randomly placing atomistic-sized 0.25 nm by 0.25 nm cuboid blocks on a regular grid in the layer, resulting in a maze-like structure, as depicted in Fig. 5 (A and B). The fraction covered by ferromagnetically coupled material is computed as *f*(*x*) = (*x* − 50)/50, with *x* being the Fe concentration in at %. This means that *x* = 75 leads to equally sized ferromagnetically and antiferromagnetically coupled regions in the spacer layer, while *x* = 50 is assumed to result in purely antiferromagnetic coupling across the spacer layer. This approach mimics the case where the Co ferromagnetic layers are separated by a two-monolayer-thick RuFe spacer layer. In this structure, atoms in the Co layer are ferromagnetically coupled across a pair of Fe atoms and antiferromagnetically coupled across either a pair of Ru atoms or across one Ru and one Fe atom.

We assume *A*_ex_(RuFe) = 21 pJ/m in the ferromagnetically coupled regions of the RuFe layer, which is similar to bulk Fe properties. Although, in our model, the ferromagnetically coupled regions in the RuFe consist of Fe atoms only, the charge transfer from the neighboring Ru atoms in the RuFe layer is expected to decrease *A*_ex_(RuFe), as observed in RuCo alloys (*17*). Thus, the calculations are also performed with the reduced *A*_ex_(RuFe) = 10.5 pJ/m.

To determine the coupling strength in the antiferromagnetically coupled regions of the RuFe layer, *J*_AF_(RuFe), we consider the averaged bilinear coupling constant

$$J_{1}(x)=[1-f(x)]J_{\text{AF}}(\text{RuFe})-f(x)A_{\text{ex}}(\text{RuFe})/d$$ (2)

where *d* is the thickness of the RuFe layer. Assuming *d* = 0.5 nm and *J*_1_(78) = 0 (from Fig. 3), 2 yields *J*_AF_(RuFe) = 53.4 mJ/m^2^ (26.7 mJ/m^2^). Similar antiferromagnetic coupling strengths are theoretically predicted in perfect Co/Ru superlattices (*28*).

In a next step, for 70 ≤ *x* ≤ 80, micromagnetic simulations are used to compute the equilibrium magnetization angle between the Co layers, θ(*x*), and Eq. 2 is used to determine *J*_1_(*x*). Then, by minimizing Eq. 1 with respect to θ, one can obtain *J*_2_(*x*) = − 0.5*J*_1_(*x*)/ cos (θ(*x*)).

The simulation results shown in Fig. 5C are in good accordance with the experimental findings (Fig. 4A). Notably, θ is accurately predicted by the model. *J*_1_ shows the same qualitative behavior, as it decreases with increasing *x* and becomes negative for *x* > 78, which is by construction of the model and thus not unexpected. *J*_2_ is well predicted with respect to its qualitative magnitude. Namely, the value for *J*_2_ is slightly underestimated for *A*_ex_(RuFe) = 10.5 pJ/m and slightly overestimated for *A*_ex_(RuFe) = 21 pJ/m, suggesting an actual ferromagnetic coupling in between the two considered values. We attribute deviations from the experimental data to our simplistic assumptions, i.e., constant *A*_ex_(RuFe) and *J*_AF_(RuFe) and linear *f*(*x*).

Furthermore, we compare the results for random and checker pattern distributions of coupling regions with *x* = 75. Although we are using the same ratio of coupling regions, *f*(75) = 0.5, the checker pattern leads to a substantially reduced *J*_2_ = 2.86 mJ/m^2^ (*J*_2_ = 3.75 mJ/m^2^ for a random distribution), resulting in an antiferromagnetic state with θ = 180^∘^ (θ = 115^∘^ for a random distribution). This result illustrates the importance of the random distribution of coupling sites, which leads to much larger antiferromagnetic and ferromagnetic coupling areas than the 0.25 nm by 0.25 nm cuboid blocks in the checker pattern. This agrees with the analytical calculations of Slonczewski (*26*), which predict that *J*_2_ increases with the lateral size of spatial fluctuations of *J*_1_ across the film’s surface.

The micromagnetic simulations also show that spatial fluctuations of *J*_1_ cause subtle nonuniformities of magnetization in the ferromagnetic layers (Fig. 5B), which are essential for establishing noncollinear alignment between ferromagnetic layers (*26*). Despite the simplistic nature of the model, the presented simulations validate the qualitative nature of the coupling mechanism and reproduce the experimental trends of *J*_1_ and *J*_2_. Furthermore, both micromagnetic simulations (Fig. 5, A and B) and the experimental results in Supplemental Data 5 suggest that the size of the spatial fluctuation is about 2 nm.

Here, we showed that a new class of magnetic spacer layers containing a nonmagnetic material (Ru) alloyed with a ferromagnetic material (Fe) can be used to precisely control noncollinear alignment between the magnetic moments of ferromagnetic layers. The observed noncollinear coupling is isotropic (Supplemental Data 6), with the strength of *J*_2_ larger than ever previously achieved. Micromagnetic simulations reveal that *J*_2_ originates from spatial fluctuations of *J*_1_ between ferromagnetic layers across our novel spacer layer. The size of spatial oscillations is about 2 nm, ensuring that noncollinear alignment will be preserved even if structure size approaches 10 nm. This will enable fabrication of rigid noncollinear magnetic structures important for applications such as STTM RAM, SOT-MRAM, STNO, and magnetic sensors. RuFe is just an example of a wide range of spacer layer materials (such as RuCo, RuMn, IrCo, and IrFe) that could be explored for controlling noncollinear alignment (*29*).

## MATERIALS AND METHODS

### Sample preparation

The studied structures, Ru_100−*x*_Fe*_x_*(18) single films and Ta(3.5)∣Ru(3.5)∣Co(*t*)∣Ru_100−*x*_Fe*_x_*(*d*)∣Co(*t*)∣Ru(3.5) multilayers, are deposited with radio frequency magnetron sputtering on (100) Si substrates at room temperature and an argon pressure below 2 mtorr. In these structures, *t* is the thickness of Co. The Ta seed layer is deposited to induce the 〈0001〉 growth orientations of the Ru, Co, and Ru_100−*x*_Fe*_x_* layers, and the top Ru film is used to protect the Co layers from oxidation.

Before deposition, (100) Si substrates are cleaned with the standard Radio Corporation of America Standard Clean (RCA SC-1) process to remove particles and organic contaminants. Clean substrates are first placed in a load lock chamber, which is evacuated to about 5×10^−7^ torr, and then transferred, without breaking the vacuum, to a process chamber with a base pressure below 5×10^−8^ torr for deposition. The films are deposited by a radio frequency magnetron sputtering from four elemental targets, 2 inches in diameter, of Ta, Ru, Co, and Fe at an argon pressure below 2 mtorr. The target-to-substrate distance is around 8 inches. The substrate holder rotates during the deposition to ensure thickness and composition uniformity of the deposited films across the substrate surface. The entire sputter process is computer controlled.

### Structural and magnetic measurements

X-ray measurements are performed using the Malvern Panalytical X’Pert Pro equipped with a CuK_α_ source. A calibration of the growth rates is inferred from fitting x-ray reflectivity measurements of single layers of each material or Ru(2)∣X∣Ru(2) (X = Co, Fe, Ta, and RuFe) multilayers with X’Pert reflectivity software from Malvern Panalytical. Large angle x-ray diffraction measurements show that multilayer structures have strong texture along the 〈0001〉 crystallographic orientations with a *c*-axis full-width-at-half-maximum distribution under 5^∘^. The single Ru_100−*x*_Fe*_x_*(18) films have hexagonal close-packed crystal structure and weak texture along the 〈0001〉 crystal directions.

The field dependence of the magnetization, *M*(*H*), is measured using a superconducting quantum interference device (SQUID) made by Quantum Design and a vibration sample magnetometer (VSM) made by Cryogenic Limited in magnetic fields up to 7 T. A magneto-optical Kerr microscope (Evico) is used to image magnetic domain structure in our magnetic films.

### Modeling of *M*(*H*) and Kerr microscopy

We used two one-dimensional micromagnetic models to fit the field dependence of the magnetization, *M*(*H*), of Co∣Ru_100−*x*_Fe*_x_*∣Co and obtain *J*_1_ and *J*_2_. The first, proposed by Eyrich *et al*. (*17*), assumes that the magnetization of Co∣Ru_100−*x*_Fe*_x_*∣Co is entirely due to the ferromagnetic Co layers. The second is a modified version of Eyrich’s model, which accounts for the magnetization of both the Co layers and the Ru_100−*x*_Fe*_x_* spacer layer. The angle between the magnetic moments of the Co layers is determined by the expression θ = 180 − arccos [*J*_1_/(2*J*_2_)].

Both models are limited to the experimentally relevant situation in which the external magnetic field is applied parallel to the surface of the films, the magnetic moments lie in the plane during magnetization reversal, and the in-plane magnetic anisotropy of the ferromagnetic layers is negligible. In addition, they presume that the saturation magnetization *M*_s_ and exchange stiffness *A*_ex_ do not vary across the ferromagnetic layers.

In the studied multilayers, the uniaxial magnetocrystalline anisotropy field of the Co layers is along the 〈0001〉 directions, perpendicular to the Co film plane. The demagnetizing dipolar field in the Co films is much larger than the uniaxial magnetocrystalline anisotropy field, forcing the magnetization to lie in the plane of the film. Because of the polycrystalline nature of the studied samples, the in-plane magnetocrystalline anisotropy is averaged. In this case, the anisotropy and demagnetization energies can be ignored in calculating the total magnetic energy, as was done in Eyrich’s model.

In the proposed models, it is assumed that each ferromagnetic layer consists of *N* sublayers that interact only with their nearest-neighbor sublayers through direct exchange interaction. The coupling across the spacer layer, RuFe in our case, is established only between the two ferromagnetic sublayers adjacent to the spacer layer. Then, in the presence of an in-plane external magnetic field, the total magnetic energy of Co∣Ru_100−*x*_Fe*_x_*∣Co per unit area, *E*_Total_, can be expressed using Eyrich’s model

$$\begin{array}{c} E_{\text{Total}}=E_{\text{RKKY}}+E_{\text{ex}}+E_{Z,\text{Co}}\\ E_{\text{RKKY}}=J_{1}\text{cos}(\theta_{N}-\theta_{N+1})+J_{2}{\text{cos}}^{2}(\theta_{N}-\theta_{N+1})\\ E_{\text{ex}}=-\frac{2A_{\text{ex}}}{a}[\overset{N-1}{\underset{i=1}{\Sigma }}\text{cos}(\theta_{i}-\theta_{i+1})+\overset{2N-1}{\underset{i=N+1}{\Sigma }}\text{cos}(\theta_{i}-\theta_{i+1})]\\ E_{Z,\text{Co}}=-{\mathrm{aM}}_{s}(\text{Co})H\overset{2N}{\underset{i=1}{\Sigma }}\text{cos}(\theta_{i}) \end{array}$$ (3)

Here, *E*_RKKY_ is the interlayer exchange coupling energy between Co sublayers *N* and *N* + 1, sandwiching the spacer layer, *E*_ex_ is the direct exchange interaction energy between nearest-neighbor sublayers within each Co layer, and *E*_*Z*,Co_ is the Zeeman energy due to the interaction between the applied field and the magnetic moments in each Co sublayer. In addition, *a* is the thickness of the Co sublayers, which we assumed to be 0.2 nm; *M_s_*(Co) is the saturation magnetization of the Co layers; *H* is the applied external magnetic field; and θ*_i_* is the angle between the magnetic moment of Co sublayer *i* and the applied external magnetic field.

As was shown in Fig. 4, the RuFe spacer layer also has a magnetic moment. Since the magnetization of RuFe is predominantly due to proximity polarization from the surrounding Co layers, we presume in our modified version of Eyrich’s model that the magnetization of RuFe is induced by Co sublayers *N* and *N* + 1. In the presence of an in-plane external magnetic field, half of the magnetization of the spacer layer reverses with sublayer *N*, and the other half reverses with sublayer *N* + 1. In this case, *E*_Total_ is identical to that of Eyrich’s model except for the addition of *E*_*Z*,RuFe_, the Zeeman energy due to the interaction between the applied field and both halves of the spacer layer. The total energy can be written as

ETotal=ERKKY+Eex+EZ,Co+EZ,RuFeEZ,RuFe=−0.5tMs(RuFe)H[cos(θN)+cos(θN+1)] (4)

where *E*_RKKY_, *E*_ex_, and *E*_*Z*,Co_ are defined in Eq. 3, and *t* and *M*_s_(RuFe) are the thickness and saturation magnetization of the RuFe spacer layer, respectively.

To fit the *M*(*H*) curves, Eq. 3 or 4 is first minimized for each Co sublayer with respect to θ*_i_* (∂E_Total_/∂θ*_i_* = 0). From this, the total magnetization along the external magnetic field direction as a function of the field strength is calculated. Both models have three fitting parameters: *J*_1_, *J*_2_, and *A*_ex_. If coupling between the Co layers is described with both *J*_1_ and *J*_2_, as in our models, then *A*_ex_ can be shown to vary with the thickness of the Co layers (*17*) from 15.5 pJ/m for *t* >7 nm to 10 pJ/m for *t* = 2 nm. To account for this variation, *M*(*H*) curves are fitted assuming the lowest and highest values of *A*_ex_ (10 and 16 pJ/m). The *J*_1_ and *J*_2_ error bars account for this uncertainty in *A*_ex_.

**Fig. 6:**
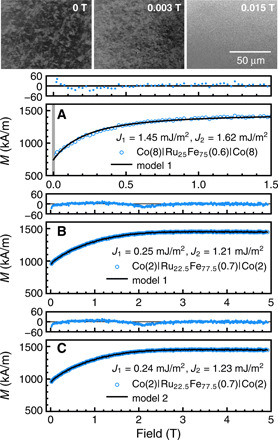
Kerr microscopy measurements and fitted *M*(*H*) curves. (**A**) *M*(*H*) curve of Co(8)∣Ru_25_Fe_75_(0.6)∣Co(8), fitted with model 1 and assuming *A*_ex_Co = 16 pJ/m. The shaded gray line represents the narrow magnetic field range in which domains were observed. The images at the top are the Kerr microscopy measurements at 0, 0.003, and 0.015 T. The *M*(*H*) curves of Co(2)∣Ru_25.5_Fe_75.5_(0.7)∣Co(2) are fitted with (**B**) model 1 and (**C**) model 2, assuming *A*_ex_(Co) = 10 pJ/m. For all *M*(*H*) plots, the empty circles are the measured values, and the solid line passing through them is the fitted curve. The plots at the top show the residuals from the fits.

Figure 6A shows the magneto-optical Kerr microscopy measurements and the *M*(*H*) curve for Co(8)∣Ru_25_Fe_75_(0.6)∣Co(8). The *M*(*H*) curve is fitted with Eyrich’s model (model 1), assuming *A*_ex_(Co) = 16 pJ/m. To reproduce our *M*(*H*) measurements, an external magnetic field of magnitude up to 0.4 T is initially applied parallel to the Co(8)∣Ru_25_Fe_75_(0.6)∣Co(8) film plane in the Kerr microscope. Subsequently, the domain structure is observed, while the field is reduced. The Kerr microscopy measurements show uniform magnetization of Co films for fields above 0.005 T. For fields below 0.005 T, the domain structure appears and remains until the field is reversed and reaches about −0.005 T. This shows that the above-discussed models correctly describe the magnetization reversal in our films over practically the entire magnetic field range (from 0.005 to 5 T).

Figure 6 (B and C) shows the *M*(*H*) curves for Co(2)∣Ru_22.5_Fe_77.5_(0.7)∣Co(2) fitted with Eyrich’s model (model 1) and the modified model (model 2), respectively. The saturation magnetization values are as follows: *M*_s_(Co) = 1450 kA/m for model 1, and *M*_s_(Co) = 1336 kA/m and *M*_s_(RuFe) = 680 kA/m for model 2. Both models fit the *M*(*H*) data well. $\chi_{\text{red}}^{2}(m\text{odel}\;1)$ = 2.78 and $\chi_{\text{red}}^{2}(m\text{odel}\;2)$ = 3.26. Furthermore, both models yield practically the same fitting parameters. For this reason and to simplify the fitting procedure, the *M*(*H*) values of all Co∣Ru_100−*x*_Fe*_x_*∣Co structures in our manuscript are analyzed using only Eyrich’s model, model 1. In Supplemental Data 7, we also showed the *M*(*H*) curves fitted with both models for films with a lower Fe concentration in the RuFe spacer layer [Co(2)∣Ru_27_Fe_73_(0.7)∣Co(2)]. Again, both models yielded practically the same fitting parameters.

It is important to point out that for θ < 45^∘^, θ is more difficult to measure since *M*_r_/*M*_s_ is proportional to cos (θ). Thus, the error bars on θ measurements increase as θ becomes smaller, as is evident in Fig. 3C. An increase in *J*_1_ and *J*_2_ causes the magnetic field required to saturate the Co magnetic moments of Co(2)∣Ru_100−*x*_Fe*_x_*(*d*)∣Co(2), *H*_s_, to also increase. For some measured structures, *H*_s_ approaches 7 T, the magnetic field available in our SQUID magnetometer and VSM. In this case, the error bars of measured *J*_1_ and *J*_2_ also increase.

## Supplementary Material

http://advances.sciencemag.org/cgi/content/full/6/48/eabd8861/DC1

Adobe PDF - abd8861_SM.pdf

Control of the noncollinear interlayer exchange coupling

## SUPPLEMENTARY MATERIALS

Supplementary material for this article is available at http://advances.sciencemag.org/cgi/content/full/6/48/eabd8861/DC1
