# Supplementary material for: Control of the noncollinear interlayer exchange coupling
Source: Sci Adv. 2020 Nov 25;6(48):eabd8861. doi: 10.1126/sciadv.abd8861 (PMC7688329; doi:10.1126/sciadv.abd8861)
Supplement: http://advances.sciencemag.org/cgi/content/full/6/48/eabd8861/DC1 [file supp_6_48_eabd8861__index.html]

Science Advances | Science AdvancesAAASSearchScience AdvancesMenu

## Supplementary Materials

# Control of the noncollinear interlayer exchange coupling

Zachary R. Nunn, Claas Abert, Dieter Suess, Erol Girt

Download Supplement

**This PDF file includes:**

- Supplemental Data 1 to 7
- Figs. S1 to S8
- References

**Files in this Data Supplement:**

- Adobe PDF - abd8861\_SM.pdf
