# Supplementary material for: Control of the noncollinear interlayer exchange coupling
Source: Sci Adv. 2020 Nov 25;6(48):eabd8861. doi: 10.1126/sciadv.abd8861 (PMC7688329; doi:10.1126/sciadv.abd8861)
Supplement: http://advances.sciencemag.org/cgi/content/full/6/48/eabd8861/DC1 [file supp_6_48_eabd8861__1.pdf]

Science Advances
AAAS

advances.sciencemag.org/cgi/content/full/6/48/eabd8861/DC1

Supplementary Materials for

Control of the noncollinear interlayer exchange coupling

Zachary R. Nunn*, Claas Abert*, Dieter Suess, Erol Girt*

*Corresponding author. Email: znunn@sfu.ca (Z.R.N.); egirt@sfu.ca (E.G.); claas.abert@univie.ac.at (C.A.)

Published 25 November 2020, *Sci. Adv.* **6**, eabd8861 (2020)
DOI: 10.1126/sciadv.abd8861

This PDF file includes:

Supplemental Data 1 to 7
Figs. S1 to S8
References

I. SUPPLEMENTAL DATA 1

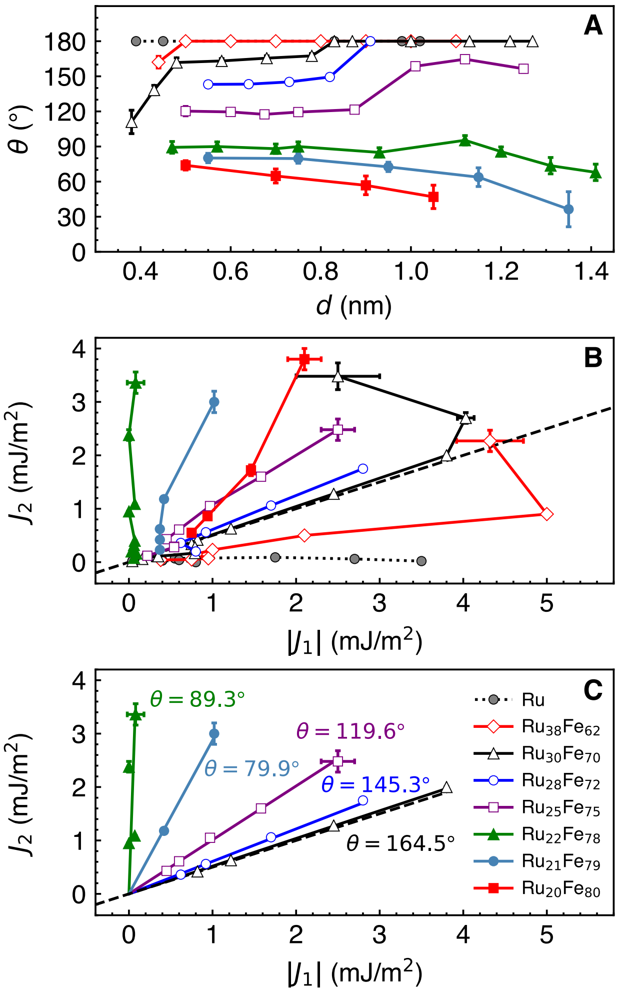

FIG. S1. **Non-collinear region determined from $J_2/|J_1|$ ratio.** (**A**) θ versus d, (**B**,**C**) J_2 versus $|J_1|$ of Co(2)|$Ru_{100-x}Fe_x(d)$|Co(2). The dashed line, $J_2 = J_1/2$, separates the regions of non-collinear coupling above the line from collinear coupling below the line. The solid lines in (C) represent the curves $J_2 = J_1(2cos(180° - \theta))^{-1}$, where θ is 79.9°, 89.3°, 119.6°, 145.3°, and 164.5°.

In Fig. 3 of our manuscript, J_1, J_2, and θ are plotted versus the thickness of the RuFe spacer layer, d, for all studied Co(2)|$Ru_{100-x}Fe_x(d)$|Co(2) structures. In these structures, the numbers in parentheses indicate the layer thicknesses in nm, x is the atomic concentration of Fe in the RuFe, and d is the thickness of the RuFe layer. The non-collinear coupling region can be directly identified from a θ versus d plot (Fig. 3C). Plotting J_2 versus $|J_1|$ can also be used to visualize the non-collinear coupling region. This is done in Fig. S1B and C. The dashed line in Fig. S1B and C is the curve $J_2 = J_1/2$, which separates the regions of non-collinear coupling above the line from the regions of collinear coupling below the line. Fig. S1A is the same as Fig. 3C in our manuscript and is used for comparison.

Of particular interest for application are Co|$Ru_{100-x}Fe_x(d)$|Co structures, where x is between about 70 and 79 at. % and $0.5 \leq d \leq 0.8$ nm. In these structures, θ depends only on x and is independent of the spacer layer thickness, as shown in Fig. S1A. This means that, in these structures, the ratio $J_2/|J_1|$ is also constant for each Fe concentration x and is equal to

$$\frac{J_2}{|J_1|} = \frac{1}{2cos(180° - \theta)}. \tag{1}$$

This linear dependence is evident from Fig. S1C, where J_2 versus $|J_1|$ is plotted for $70 \leq x \leq 79$ and $0.5 \leq d \leq 0.8$ nm. The solid lines in Fig. S1C represent the curves calculated using (1), where θ is 79.9°, 89.3°, 119.6°, 145.3°, and 164.5°. Here, θ is the average coupling angle for each x in the spacer layer thickness range $0.5 \leq d \leq 0.8$ nm.

II. SUPPLEMENTAL DATA 2

Figure S2 shows the saturation magnetization of Co(2)|$Ru_{100-x}Fe_x(t)$|Co(2) structures for $t = 0.7$ and 1.2 nm and $0 \leq x \leq 82$.

III. SUPPLEMENTAL DATA 3

In the studied structures, the measured J_2 always favours a perpendicular alignment and has a strength comparable to J_1, suggesting an extrinsic source *(4)*. Extrinsic sources of J_2 could be pin-holes *(24)*, uncorrelated

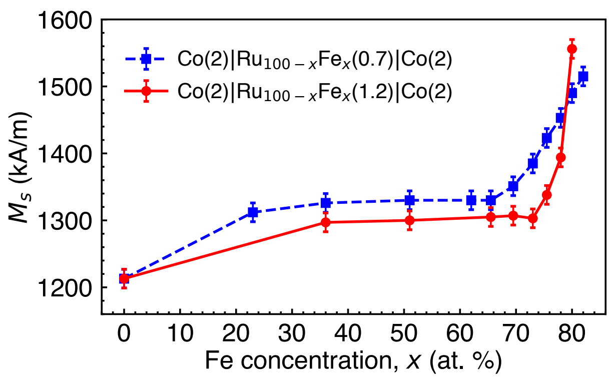

FIG. S2. **Saturation magnetization of Co|RuFe|Co.** The dependence of the saturation magnetization, M_s, of Co(2)|$Ru_{100-x}Fe_x(t)$|Co(2), for $t = 0.7$ and 1.2 nm, on Fe concentration, x, at 298 K. This figure shows M_s over a wider x range than Fig. 4B.

film roughness *(4)*, loose spins *(25)*, and spatial fluctuations *(26)*. It is shown in the manuscript that spatial fluctuations are the source of the large J_2 in our multilayers. In this section and in Supplemental Data 4, we will discuss the first three mechanisms in detail.

Pin-holes: Fe is soluble over a large composition range in Ru; thus, the existence of pin-holes in our films is not expected. Microstructures of Co|Ru multilayers have been extensively studied in both academia *(30)* and industry *(7)*, and the presence of pin-holes has not been reported.

Uncorrelated film roughness: When the film roughness is uncorrelated, the orange-peel coupling is zero, and the biquadratic coupling contribution can be estimated from a simple model that assumes one smooth ferromagnetic/spacer layer interface and the other interface with roughness described by the sinusoidally varying function $\delta \cdot cos(2\pi x/L)$. J_2 can then be calculated as *(4)*

$$J_2 \sim \frac{\mu_0 M_s^4 L \delta^2}{A(\mathrm{Co})} e^{-4\pi D/L}. \qquad (2)$$

The period and the root mean square roughness of our films are $L \sim 30$ nm and $\delta = 0.12$ nm, respectively. The thickness of our spacer layers is roughly $D = 0.7$ nm, with the exchange stiffness of 2 nm Co layers being $A_{\mathrm{ex}}(\mathrm{Co}) = 13$ pJ/m. The roughness-induced biquadratic coupling is then $J_2 = 0.1\,\mathrm{mJ/m^2}$. This is forty times smaller than the largest measured J_2 in our films.

L and δ are determined from atomic force microscopy (AFM) measurements of Sample 1, Ta(3)|Ru(3.5)|Co(2)|$Ru_{100-x}Fe_x$(0.7), and Sample 2, Ta(3)|Ru(3.5)|Co(2)|$Ru_{100-x}Fe_x$(0.7)|Co(2)|Ru(3.5). AFM measurements are performed in non-contact mode using NANOSENSORS PPP-NCHR-10 probes with a typical tip radius of less than 7 nm. L is found to be about 30 nm in both samples and δ is found to be 0.12 nm for Sample 1 and 0.1 nm for Sample 2. AFM measurements of Sample 1 over $1\,\mu\mathrm{m} \times 1\,\mu\mathrm{m}$ and $400\,\mathrm{nm} \times 400\,\mathrm{nm}$ film surfaces are shown in Fig. S3.

Loose spins: The loose spin model was developed to explain a strong temperature dependence of biquadratic coupling in structures where two ferromagnetic layers are coupled across a nonmagnetic layer doped with magnetic impurities *(25)*. These magnetic impurities are assumed to be paramagnetic and weakly coupled to the surrounding ferromagnetic layers by indirect exchange coupling. In the presented non-collinear structures, the coupling is mediated by magnetic spacer layers and the magnetic impurities are strongly coupled by predominantly direct exchange interaction to the adjacent Co layers. Thus, this model is not appropriate to explain the presence of the large J_2 in our films. This is further confirmed in Supplemental Data 4 by showing that the temperature dependence of J_1 and J_2 in the studied non-collinear structures can be explained by the spatial fluctuation model *(26)* and not by the loose spin model *(25)*.

IV. SUPPLEMENTAL DATA 4

To further shed light on the origin of J_2, a study of the temperature dependence of J_1, J_2, θ, and M_s of Co(2)|$Ru_{31}Fe_{69}$(0.7)|Co(2) and

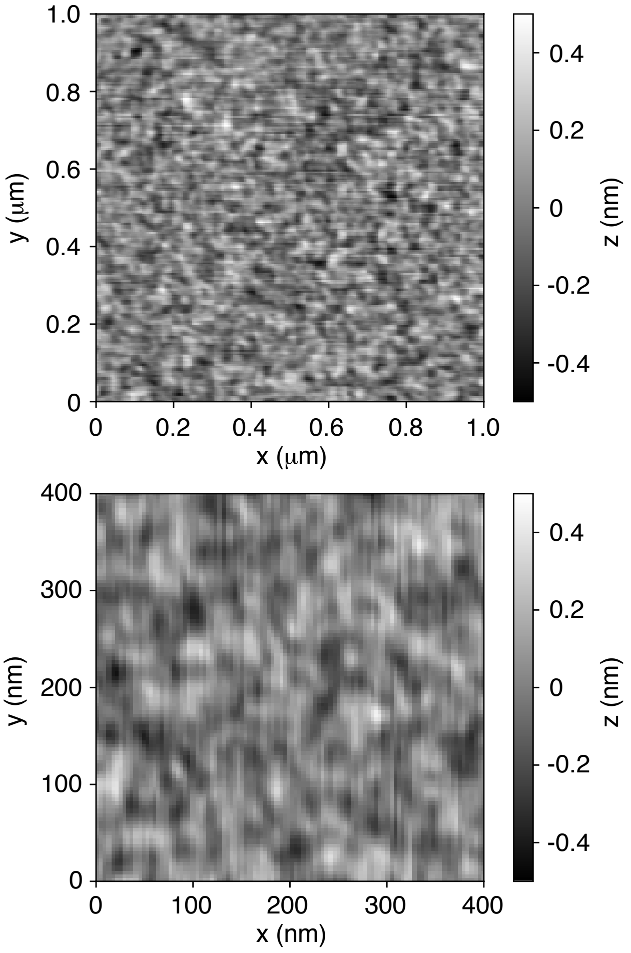

FIG. S3. AFM measurements. Sample 1: Ta(3)|Ru(3.5)|Co(2)|$Ru_{100-x}Fe_x$(0.7).

Co(2)|$Ru_{20}Fe_{80}$(0.7)|Co(2) is performed with SQUID (Fig. S4). These two multilayer structures are chosen because the Fe concentrations in the RuFe layers are close to the minimum and maximum concentrations required for non-collinear coupling in Co|RuFe|Co (non-collinear coupling in Co|$Ru_{100-x}Fe_x$|Co occurs for $68 \leq x \leq 82$ at. %: Fig. 4A). Furthermore, in these two structures, the RuFe spacer layer has different magnetic properties. In the first structure, the presence of adjacent Co layers induces a small magnetic moment in the $Ru_{31}Fe_{69}$ layer (166 kA/m at 298 K). In the second structure, the $Ru_{20}Fe_{80}$ layer has a large magnetic moment (983 kA/m at 298 K) that, in part, persists even in the absence of the surrounding Co layers (the magnetic moment of the single $Ru_{20}Fe_{80}$ layer is equal to 130 kA/m). This difference in the magnetic properties of the RuFe spacer layers could affect the mechanism responsible for large J_2 in Co|RuFe|Co. For comparison, M_s of a single Co(4) layer is also measured from 298 to 10 K.

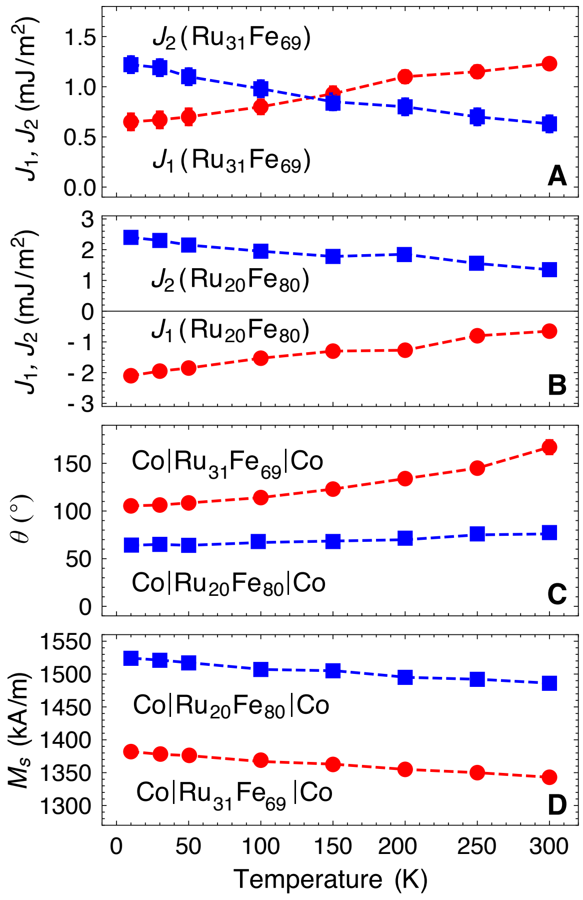

FIG. S4. Temperature dependence of magnetic parameters. Temperature dependence of (**A**,**B**) J_1, J_2, (**C**) θ, and (**D**) M_s of Co(2)|$Ru_{100-x}Fe_x$(0.7)|Co(2), where $x = 69$ and 80 at. %. For the majority of the measurements, the error bars are smaller than the marker size. Dashed lines are a guide to the eye.

Figure S4 shows that, in both structures, J_1 decreases and J_2 increases with decreasing temperature from 298 to 10 K. Additionally, in both structures, θ decreases with decreasing temperature as $\theta = 180° - a\cos(J_1/(2J_2))$, and M_s increases at lower temperatures.

The M_s of a single Co(4) layer increases from 1314 to 1338 kA/m as the temperature is reduced from 298

to 10 K (Fig. 4). Assuming that the M_s of Co in Co|$Ru_{31}Fe_{69}$|Co and Co|$Ru_{20}Fe_{80}$|Co has the same temperature dependence, one can calculate the $M_s(T)$ of the $Ru_{31}Fe_{69}$ and $Ru_{20}Fe_{80}$ spacer layers by taking the difference of the $M_s(T)$ of the Co|$Ru_{31}Fe_{69}$|Co (or Co|$Ru_{20}Fe_{80}$|Co) and the $M_s(T)$ of the Co layers. This results in an increase in the M_s of $Ru_{31}Fe_{69}$ in Co|$Ru_{31}Fe_{69}$|Co from 166 kA/m at 298 K to 251 kA/m at 10 K and of $Ru_{20}Fe_{80}$ in Co|$Ru_{20}Fe_{80}$|Co from 983 kA/m at 298 K to 1063 kA/m at 10 K. Thus, the increase in M_s of Co|RuFe|Co with the reduction in temperature (Fig. S4D) seems to be predominantly due to the increase of the saturation magnetization of the RuFe spacer layers in these multilayers.

Spatial fluctuations: The spatial fluctuation mechanism can qualitatively explain the temperature dependence of J_1 and J_2 of both samples in Fig. S4A and B. Let's assume that, in Co|RuFe|Co, the sign of the interlayer exchange coupling varies spatially. M_s of the RuFe spacer layer increases as the temperature is reduced, suggesting that the ferromagnetic coupling across the RuFe also increases. This could be due to increase of the exchange stiffness and/or the surface fraction of the spatial areas mediating ferromagnetic coupling. Antiferromagnetic coupling in Co|Ru|Co is also known to increase as the ambient temperature decreases *(31, 32)*. Thus, the reduction of temperature is expected to lead to an increase in the strength of both ferromagnetic and antiferromagnetic coupling in Co|RuFe|Co. This should lead to an increase in the magnitude of spatial fluctuations of J_1, ΔJ_1, and thus to an increase in J_2, in agreement with the results in Fig. S4. The reduction of J_1 in Fig. S4 can be explained by a larger increase of ferromagnetic coupling than antiferromagnetic coupling in Co|RuFe|Co at lower temperatures.

Loose spins: In this model, the exchange coupling constants are calculated as

$$\begin{aligned} J_1(T) &= 0.5\ \rho\ [f(T,\pi) - f(T,0)] \\ J_2(T) &= \rho\ [0.5\ f(T,0) + 0.5\ f(T,\pi) - f(T,\pi/2)], \end{aligned} \tag{3}$$

where $\rho = c/(\sqrt{3}/2\ a^2)$ is the areal density of the loose spins, c is the fractional concentration of loose spins, $a = 2.51 \times 10^{-10}$ m, and $f(T,\theta)$ is the free energy per loose spin given by

$$f(T,\theta) = -k_B T\ ln\left(\frac{\sinh\{[1+(2S)^{-1}]U(\theta)/(k_B T)\}}{\sinh\{U(\theta)/(2Sk_B T)\}}\right). \tag{4}$$

In (4), $U(\theta) = [U_1^2 + U_2^2 + 2U_1U_2\ \cos(\theta)]^{0.5}$, U_1 and U_2 are exchange coupling fields on a loose spin from the surrounding ferromagnetic layers, θ is the angle between magnetic moments of surrounding ferromagnetic layers, k_B is the Boltzmann constant, and S is the spin quantum number, which we assume to be 1 in our calculations *(25)*.

The loose spin model is used to fit the temperature dependence of J_1 and J_2 of Co|$Ru_{31}Fe_{69}$|Co. In this film structure, two ferromagnetic layers are coupled across a weakly magnetic spacer layer (not nonmagnetic as assumed in the model). However, since $Ru_{31}Fe_{69}$ is nonmagnetic in the absence of Co layers, it is still important to test whether the loose spin model can explain the measured temperature dependence of the coupling constants in this structure.

Fe magnetic impurities are randomly distributed in the $Ru_{31}Fe_{69}$ spacer layer. Thus, it is first assumed that $U_1 = U_2$ and $c = 0.69$. In this case, the dependence of J_2 on temperature, $J_2(T)$, is nearly exponential and does not follow $J_2(T)$ of Co|$Ru_{31}Fe_{69}$|Co, as shown in Fig. S5B. $J_1(T)$ is also calculated using values of $U_{1,2}$ obtained from fitting $J_2(T)$ (Fig. S5A).

The $Ru_{31}Fe_{69}$ spacer layer is several atomic layers thick; thus, the exchange coupling fields from the top and bottom magnetic layers on magnetic impurities in

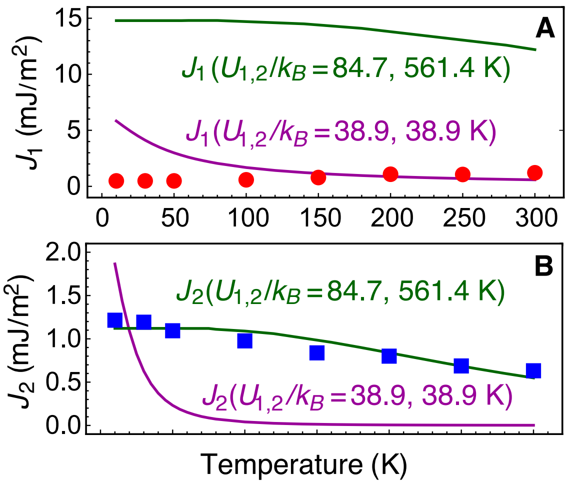

FIG. S5. Fitting $J_1(T)$ and $J_1(T)$ with the loose spin model. Measured and calculated/fitted values of (**A**) J_1 and (**B**) J_2 of Co|$Ru_{31}Fe_{69}$|Co as a function of temperature. $J_2(T)$ is fitted using the loose spin model and assuming $c = 0.69$ and either 1) $U_1 = U_2$ or 2) $|U_1| \neq |U_2|$. $J_1(T)$ is calculated using values of U_1 and U_2 obtained from fit of $J_2(T)$. $J_1(T)$ and $J_2(T)$ cannot be simultaneously fitted using the loose spin model. The error bars are smaller than the marker size.

each atomic layer may be different. We will assume that $Ru_{31}Fe_{69}$ is two atomic layers thick. Due to symmetry arguments, $U_1 = U_{1T} = U_{2B}$ and $U_2 = U_{1B} = U_{2T}$, where U_{1T} and U_{1B} are the exchange coupling fields from the bottom magnetic layer on the top and bottom atomic layers of the spacer layer, respectively, and U_{2T} and U_{2B} are the exchange coupling fields from the top magnetic layer on the top and bottom atomic layers of the spacer layer, respectively. Thus, $J_2(T)$ is also fitted assuming $|U_1| \neq |U_2|$ and $c = 0.69$.

Fig. S5B shows reasonable agreement between the experimental data for $J_2(T)$ and the fit with fitting parameters $U_1/k_B = 84.7\,\text{K}$ and $U_2/k_B = 561.4\,\text{K}$. Unfortunately, $J_1(T)$ calculated using the values of $U_{1,2}$ obtained from fitting $J_2(T)$ has a different trend and is about an order of magnitude larger than the experimentally obtained $J_1(T)$ (Fig. S5A).

Additionally, the experimental data for $J_1(T)$ and $J_2(T)$ of Co|$Ru_{31}Fe_{69}$|Co cannot be simultaneously fitted using the loose spin model and assuming $c = 0.69$ and either $U_1 = U_2$ or $|U_1| \neq |U_2|$. Thus, the loose spin mechanism cannot explain the temperature behaviour of the coupling constants of Co|$Ru_{31}Fe_{69}$|Co and is not the mechanism responsible for the large J_2 in this sample.

V. SUPPLEMENTAL DATA 5

The size of spatial fluctuations. The spatial fluctuation mechanism *(26)* is based on a magnitude change of the bilinear coupling term, J_1, across the film's plane. These fluctuations are usually due to a spatial variation of the spacer layer thickness *(33, 34)*. The competition between the coupling and stiffness energies leads to an orthogonal (90°) alignment between the magnetic moments. Following the theory proposed by Slonczewski *(26)*, the strength of the biquadratic coupling between two identical magnetic layers of thickness t across a spacer layer can be determined from the following relation:

$$J_2 = \frac{4(\Delta J_1)^2 L}{\pi^3 A(\text{Co})} \coth\left(\pi \frac{t}{L}\right), \tag{5}$$

where $A_{\text{ex}}(\text{Co})$ is the exchange stiffness of the Co layers and ΔJ_1 and L are the magnitude change and size of spatial fluctuations of J_1 across the film's surface, respectively.

The non-collinear structures studied in this article have spatial fluctuations ΔJ_1 originating from the random distribution of magnetic atoms in a RuFe spacer layer. As an example, this can be visualized in Co|$Ru_{25}Fe_{75}$|Co, where ferromagnetic Co layers are separated by a two monolayer thick magnetic $Ru_{25}Fe_{75}$ spacer layer. In this structure, atoms in the Co layer are ferromagnetically coupled across a pair of Fe atoms and antiferromagnetically coupled across either a pair of Ru atoms or one Ru and one Fe atom. Since the atoms in $Ru_{25}Fe_{75}$ are homogeneously distributed, it is expected that the size of spatial fluctuations, L, is very small: approaching atomic

distances. From (5), if L is less than the magnetic layer thickness t, $\coth(\pi \frac{t}{L}) = 1$ and, thus, J_2 is independent of t. Fig. S6 shows the measured and fitted $M(H)$ curves of Co(t)|$Ru_{27}Fe_{73}$(0.7)|Co(t) ($t = 1.5$, 2, 3.8, 6 nm). It is found that the structures with $t = 2$, 3.8, and 6 nm have similar J_1 and J_2 values, and that J_1 and J_2 decrease in the structure with $t = 1.5$ nm. Due to J_2 being independent of t, for $t > 2$, the size of the spatial fluctuations, L, in (5) should be about 2 nm. This is in agreement with the micromagnetic modelling results in Fig. 5A and B, which also predict that the size of spatial fluctuation is below 2 nm. This is promising for nanometer sized devices, as 2 nm is well below the current lithography process node size used in the fabrication of spintronic devices. x

VI. SUPPLEMENTAL DATA 6

The interlayer exchange coupling between two ferromagnetic layers across the majority of 3d, 4d, and 5d nonmagnetic metallic spacer layers oscillates between antiferromagnetic and ferromagnetic as a function of the spacer layer thickness *(2)*. The period of oscillation and strength of the exchange coupling depends on the crystal growth orientation of the ferromagnetic|spacer layer|ferromagnetic film structures. However, for films grown along the same crystal orientation, the bilinear coupling constant J_1 is isotropic with respect to the direction of magnetization in the ferromagnetic layers. For example, the dependence of J_1 on the spacer layer thickness in Co|Ru|Co, grown along the $\langle 0001 \rangle$ crystal orientations, is the same for magnetizations in Co layers parallel (Fig. 3A) and perpendicular to the film surface *(35)*. The origin of the large J_2 in our structures is the spatial fluctuation of J_1. J_1 is isotropic and, thus, J_2 is also expected to be isotropic.

In our manuscript, we have studied Co|RuFe|Co structures with Co layers having in-plane magnetization. To show that the coupling across RuFe is independent of the direction of magnetization in the mag-

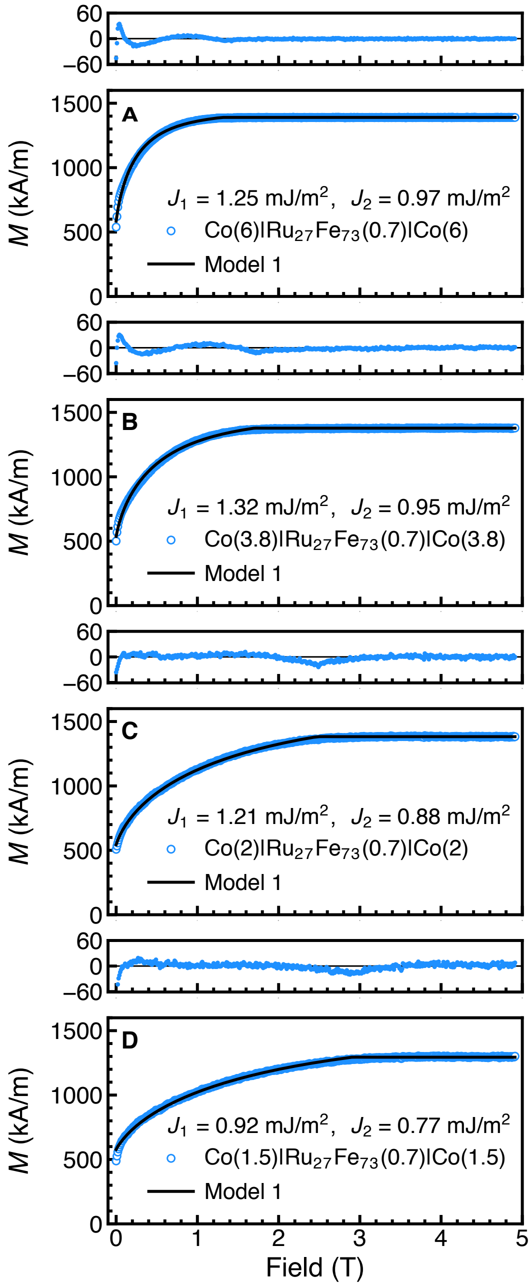

FIG. S6. $M(H)$ curves fitted with Model 1. The measured and fitted $M(H)$ curves of Co(t)|$Ru_{27}Fe_{73}$(0.7)|Co(t) for t equal to (**A**) 6 nm, (**B**) 3.8 nm, (**C**) 2 nm, and (**D**) 1.5 nm. $M(H)$ is fitted with Model 1, assuming that A_{ex}(Co) = 13 pJ/m for $t \geq 2$ nm and A_{ex}(Co) = 8 pJ/m for $t = 1.5$ nm. The empty circles are the measured values and the solid line passing through them is the fitted curve. The plots at the top show the residuals from the fit.

netic layers, we designed a film structure with two magnetic layers: one with magnetization in the film plane and the other with magnetization tilted with respect to the film normal, as in Fig. 2B. This is achieved with the $FM_1|SL|FM_2$ structure using FM_1 = Co(3)|Ru(0.8)|Co(3), SL = $Ru_{100-x}Fe_x$(0.6), and FM_2 = Co(0.25)|4×[Co(0.25)/Ni(0.6)]. In this structure, the RuFe spacer layer is surrounded with Co layers to achieve the same exchange coupling behaviour as in the three layer structures studied in the paper. $FM_1|SL|FM_2$ is grown on top of Ta(3)|Ru(3) seed layers, setting the texture of $FM_1|SL$ along $\langle 0001 \rangle$ and the texture of the FM_2 multilayer along $\langle 111 \rangle$ crystal orientations. The film structure is covered with 3 nm thick Ru for protection.

FM_1 consists of two Co layers antiferromagnetically coupled across a Ru spacer layer. The shape anisotropy field in Co films is much larger than the uniaxial magnetocrystalline anisotropy field, forcing the magnetization to lie in the plane of the film. FM_2 has perpendicular magnetic anisotropy; however, its magnetization is tilted with respect to the film normal due to non-collinear coupling with FM_1 across SL. The total intrinsic magnetic anisotropy of FM_2 is perpendicular to the plane and is adjusted to be only slightly larger than the easy-plane dipolar (shape) magnetic anisotropy of FM_2 *(36, 37)*. The magnetic anisotropy energy of FM_1 is much larger than the magnetic anisotropy energy of FM_2; thus, the exchange coupling between these two layers does not tilt the magnetization of FM_1 away from the film plane. The coupling angle between FM_1 and FM_2 is controlled by varying the concentration of Fe in SL.

Figure S7 shows polar Kerr measurements of $FM_1|SL|FM_2$. A polar Kerr magnetometer senses magnetization perpendicular to the film plane. Thus, the $M(H)$ loops in Fig. S7 represent the magnetization of FM_2 as a function of the external field. Fe concentration in $Ru_{100-x}Fe_x$, x, is initially set to 79 at. % to ensure that the magnetization of FM_2 is perpendicular to the film surface: $\theta = 90°$. The coupling angle is then increased from 90° to about 155° (the magnetization of FM_2 is tilted 65° from the film normal) by decreasing x from 79 to 71 at. %. θ is estimated from the re-

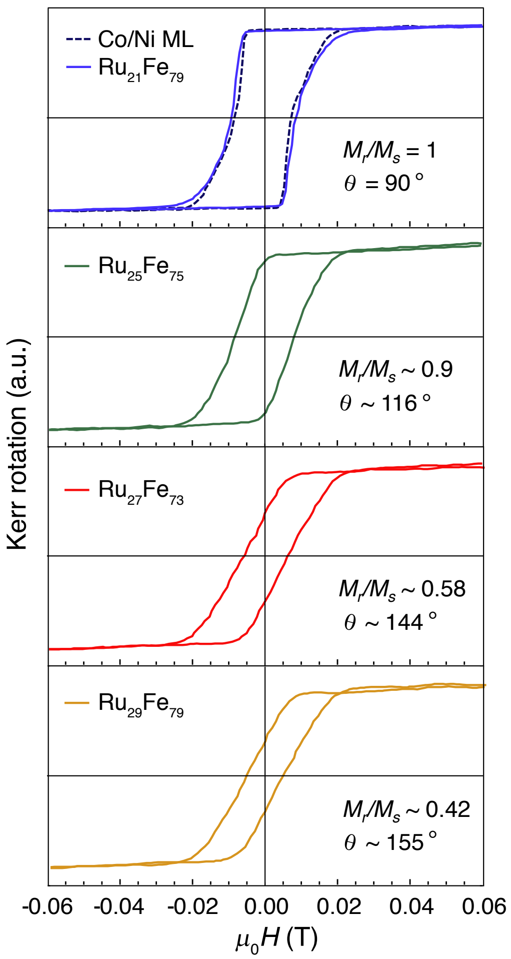

FIG. S7. Using polar Kerr measurements to determine θ. Polar Kerr measurements of $M(H)$ loops of Co(3)|Ru(0.8)|Co(3)|$Ru_{100-x}Fe_x$(0.6)| Co(0.25)|4×[Co(0.25)/Ni(0.6)]. Magnetic moments of Co layers in Co(3)|Ru(0.8)|Co(3) are parallel with the film plane and antiferromagnetically coupled across the Ru spacer layer. The Co(0.25)|4×[Co(0.25)/Ni(0.6)] layer has a perpendicular magnetic anisotropy; however, its magnetization is tilted with respect to the film normal due to non-collinear coupling with Co|Ru|Co across $Ru_{100-x}Fe_x$. The coupling angle is varied from 90° to about 155° by changing the Fe concentration, x, in the $Ru_{100-x}Fe_x$ from 79 to 71 at. %. The dashed line represents a polar Kerr measurement of $M(H)$ of only Co(0.25)|4×[Co(0.25)/Ni(0.6)].

manence (M_r) to saturation (M_s) magnetization ratio, M_r/M_s. A polar Kerr measurement of the $M(H)$ loop of only FM_2 (without FM_1|SL) is also included in Fig. S7 for comparison. Figure S7 clearly shows that varying Fe concentration in RuFe can be used to control the direction of the magnetization of FM_2. Furthermore, for the same Fe concentration in the RuFe spacer layer, the same coupling angle is obtained in both Co|RuFe|Co (with in-plane magnetizations in the Co layers) and FM_1|SL|FM_2. The isotropic nature of the interlayer coupling is important for many applications, not limiting the design of magnetic film structures, as discussed in Fig. 2.

VII. SUPPLEMENTAL DATA 7

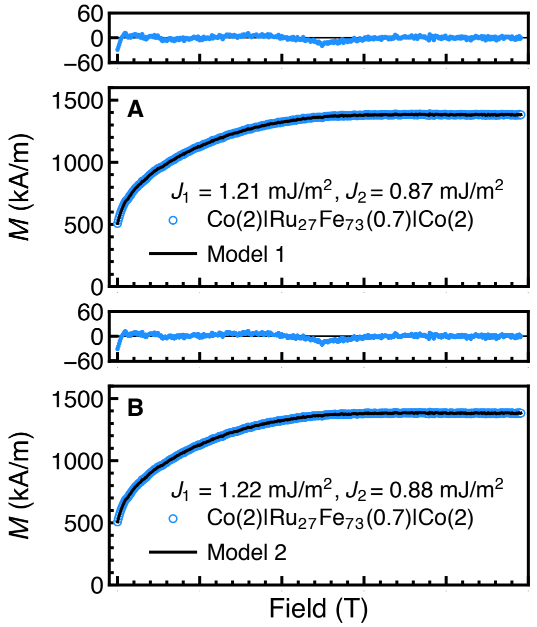

FIG. S8. ***M*(*H*) curves fitted with Model 1 and 2.** The $M(H)$ curves of Co(2)|$Ru_{27}Fe_{73}$(0.7)|Co(2) are fitted with (**A**) Model 1 and (**B**) Model 2, assuming $A_{\mathrm{ex}}(\mathrm{Co}) = 10\,\mathrm{pJ/m}$. The empty circles are the measured values and the solid line passing through them is the fitted curve. The plots at the top show the residuals from the fits.

Figure S8 shows the $M(H)$ curves for Co(2)|$Ru_{27}Fe_{73}$(0.7)|Co(2) fitted with Eyrich's model (Model 1) and the modified model (Model 2), respectively. The saturation magnetization values are: $M_s(\mathrm{Co}) = 1382\,\mathrm{kA/m}$ for Model 1, and $M_s(\mathrm{Co}) = 1326\,\mathrm{kA/m}$ and $M_s(\mathrm{RuFe}) = 320\,\mathrm{kA/m}$ for Model 2. Both models fit the $M(H)$ data well. χ^2_{red}(Model 1) = 3.63 and χ^2_{red}(Model 2) = 4.12. Furthermore, both models yield practically the same fitting parameters.

REFERENCES AND NOTES

1. P. Grünberg, R. Schreiber, Y. Pang, M. B. Brodsky, H. Sowers, Layered magnetic structures: Evidence for antiferromagnetic coupling of Fe layers across Cr interlayers. *Phys. Rev. Lett.* **57**, 2442–2445 (1986).

2. S. S. P. Parkin, Systematic variation of the strength and oscillation period of indirect magnetic exchange coupling through the 3d, 4d, and 5d transition metals. *Phys. Rev. Lett.* **67**, 3598–3601 (1991).

3. K. B. Hathaway, Magnetic coupling and magnetoresistance, in *Ultrathin Magnetic Structures II*, J. A. C. Bland, B. Heinrich, Eds. (Springer, 1994), pp. 45–194.

4. M. D. Stiles, Interlayer exchange coupling, in *Ultrathin Magnetic Structures III*, J. A. C. Bland, B. Heinrich, Eds. (Springer Berlin Heidelberg, 2005), pp. 99–142.

5. R. A. Duine, K.-J. Lee, S. S. P. Parkin, M. D. Stiles, Synthetic antiferromagnetic spintronics. *Nat. Phys.* **14**, 217–219 (2018).

6. P. Omelchenko, B. Heinrich, E. Girt, Measurements of interlayer exchange coupling of Pt in Py|Pt|Py system. *Appl. Phys. Lett.* **113**, 142401 (2018).

7. E. E. Fullerton, J. R. Childress, Spintronics, magnetoresistive heads, and the emergence of the digital world. *Proc. IEEE* **104**, 1787–1795 (2016).

8. R. Sbiaa, Magnetization switching by spin-torque effect in off-aligned structure with perpendicular anisotropy. *J. Phys. D Appl. Phys.* **46**, 395001 (2013).

9. R. Matsumoto, H. Arai, S. Yuasa, H. Imamura, Spin-transfer-torque switching in a spin-valve nanopillar with a conically magnetized free layer. *Appl. Phys. Express* **8**, 063007 (2015).

10. Y. Zhou, C. Zha, S. Bonetti, J. Persson, J. Åkerman, Spin-torque oscillator with tilted fixed layer magnetization. *Appl. Phys. Lett.* **92**, 262508 (2008).

11. A. D. Kent, D. C. Worledge, A new spin on magnetic memories. *Nat. Nanotechnol.* **10**, 187–191 (2015).

12. J. C. Slonczewski, Current-driven excitation of magnetic multilayers. *J. Magn. Magn. Mater.* **159**, L1–L7 (1996).

13. S. Mangin, D. Ravelosona, J. A. Katine, M. J. Carey, B. D. Terris, E. E. Fullerton, Current-induced magnetization reversal in nanopillars with perpendicular anisotropy. *Nat. Mater.* **5**, 210–215 (2006).

14. S. I. Kiselev, J. C. Sankey, I. N. Krivorotov, N. C. Emley, R. J. Schoelkopf, R. A. Buhrman, D. C. Ralph, Microwave oscillations of a nanomagnet driven by a spin-polarized current. *Nature* **425**, 380–383 (2003).

15. M. Cubukcu, O. Boulle, M. Drouard, K. Garello, C. O. Avci, I. M. Miron, J. Langer, B. Ocker, P. Gambardella, G. Gaudin, Spin-orbit torque magnetization switching of a three-terminal perpendicular magnetic tunnel junction. *Appl. Phys. Lett.* **104**, 042406 (2014).

16. P. Franke, D. Neuschütz, Thermodynamic properties of inorganic materials: Binary Systems. Part 5: Binary Systems Supplement 1, in *Landolt-Börnstein: Numerical Data and Functional Relationships in Science and Technology: Group IV: Physical Chemistry* (2007), vol. 19B5.

17. C. Eyrich, A. Zamani, W. Huttema, M. Arora, D. Harrison, F. Rashidi, D. Broun, B. Heinrich, O. Mryasov, M. Ahlberg, O. Karis, P. E. Jönsson, M. From, X. Zhu, E. Girt, Effects of substitution on the exchange stiffness and magnetization of Co films. *Phys. Rev. B* **90**, 235408 (2014).

18. S. S. P. Parkin, N. More, K. P. Roche, Oscillations in exchange coupling and magnetoresistance in metallic superlattice structures: Co/Ru, Co/Cr, and Fe/Cr. *Phys. Rev. Lett.* **64**, 2304–2307 (1990).

19. T. Saerbeck, N. Loh, D. Lott, B. P. Toperverg, A. M. Mulders, A. F. Rodríguez, J. W. Freeland, M. Ali, B. J. Hickey, A. P. J. Stampfl, F. Klose, R. L. Stamps, Spatial fluctuations of loose spin coupling in CuMn/Co multilayers. *Phys. Rev. Lett.* **107**, 127201 (2011).

20. M. E. Filipkowski, J. J. Krebs, G. A. Prinz, C. J. Gutierrez, Giant near-90° coupling in epitaxial CoFe/Mn/CoFe sandwich structures. *Phys. Rev. Lett.* **75**, 1847–1850 (1995).

21. T. Diederich, S. Couet, R. Röhlsberger, Noncollinear coupling of iron layers through native iron oxide spacers. *Phys. Rev. B* **76**, 054401 (2007).

22. B. Heinrich, J. F. Cochran, T. Monchesky, R. Urban, Exchange coupling through spin-density waves in Cr(001) structures: Fe-whisker/Cr/Fe(001) studies. *Phys. Rev. B* **59**, 14520–14532 (1999).

23. J. M. D. Coey, *Magnetism and Magnetic Materials* (Cambridge Univ. Press, 2010).

24. J. F. Bobo, H. Kikuchi, O. Redon, E. Snoeck, M. Piecuch, R. L. White, Pinholes in antiferromagnetically coupled multilayers: Effects on hysteresis loops and relation to biquadratic exchange. *Phys. Rev. B* **60**, 4131–4141 (1999).

25. J. C. Slonczewski, Origin of biquadratic exchange in magnetic multilayers (invited). *J. Appl. Phys.* **73**, 5957–5962 (1993).

26. J. C. Slonczewski, Fluctuation mechanism for biquadratic exchange coupling in magnetic multilayers. *Phys. Rev. Lett.* **67**, 3172–3175 (1991).

27. C. Abert, L. Exl, F. Bruckner, A. Drews, D. Suess, magnum.fe: A micromagnetic finite-element simulation code based on FEniCS. *J. Magn. Magn. Mater.* **345**, 29–35 (2013).

28. S. Zoll, A. Dinia, D. Stoeffler, M. Gester, H. A. M. van den Berg, K. Ounadjela, Preserved interfacial magnetism and giant antiferromagnetic exchange coupling in Co/Rh sandwiches. *EPL* **39**, 323–328 (1997).

29. Z. R. Nunn, E. Girt, Applications of non-collinearly coupled magnetic layers. U.S. Patent 10,204,671 (2019).

30. Y. Lee, J. Hayakawa, S. Ikeda, F. Matsukura, H. Ohno, Giant tunnel magnetoresistance and high annealing stability in CoFeB/MgO/CoFeB magnetic tunnel junctions with synthetic pinned layer. *Appl. Phys. Lett.* **89**, 042506 (2006).

31. Z. Zhang, L. Zhou, P. Wigen, K. Ounadjela, Temperature dependence of interlayer exchange coupling in Co/Ru/Co trilayer structures. *J. Appl. Phys.* **75**, 6434–6436 (1994).

32. B. Khodadadi, J. B. Mohammadi, J. M. Jones, A. Srivastava, C. Mewes, T. Mewes, C. Kaiser, Interlayer exchange coupling in asymmetric Co−Fe/Ru/Co−Fe trilayers investigated with broadband temperature-dependent ferromagnetic resonance. *Phys. Rev. Appl.* **8**, 014024 (2017).

33. B. Heinrich, Z. Celinski, J. F. Cochran, A. S. Arrott, K. Myrtle, S. T. Purcell, Bilinear and biquadratic exchange coupling in bcc Fe/Cu/Fe trilayers: Ferromagnetic-resonance and surface magneto-optical Kerr-effect studies. *Phys. Rev. B* **47**, 5077–5089 (1993).

34. S. O. Demokritov, Biquadratic interlayer coupling in layered magnetic systems. *J. Phys. D Appl. Phys.* **31**, 925–941 (1998).

35. E. Girt, H. J. Richter, Antiferromagnetically coupled perpendicular recording media. *IEEE Trans. Magn.* **39**, 2306–2310 (2003).

36. M. Arora, R. Hübner, D. Suess, B. Heinrich, E. Girt, Origin of perpendicular magnetic anisotropy in Co/Ni multilayers. *Phys. Rev. B* **96**, 024401 (2017).

37. M. Arora, N. R. Lee-Hone, T. Mckinnon, C. Coutts, R. Hübner, B. Heinrich, D. M. Broun, E. Girt, Magnetic properties of Co/Ni multilayer structures for use in STT-RAM. *J. Phys. D Appl. Phys.* **50**, 505003 (2017).
